# Particle Agglomeration and Properties of Pregelatinized Potato Starch Powder

**DOI:** 10.3390/gels9020093

**Published:** 2023-01-20

**Authors:** Hyunwoo Lee, Byoungseung Yoo

**Affiliations:** Department of Food Science and Biotechnology, Dongguk University-Seoul, Goyang 410-820, Republic of Korea

**Keywords:** pregelatinized starch, particle agglomeration, structural change, maltodextrin, rheological property

## Abstract

Pregelatinized starches are used as thickeners in many instant food products. The unique properties of pregelatinized starches, such as their dispersibility in water and high viscosity, are generally desirable for instant food products. However, powdered starches cannot be easily dispersed in cold water due to clumping. The most reliable method to solve this problem is particle size enlargement by an agglomeration technique that causes a structural change in the starch. In this study, pregelatinized potato starch powder (PPSP) was agglomerated in a fluidized bed agglomerator, after which the physical, structural, and rheological properties of the PPSP agglomerated with different maltodextrin (MD) binder concentrations were investigated. The powder solubility and flowability (CI and HR) of all the agglomerated PPSPs were improved, and the particle size (D_50_) tended to increase as the MD concentration increased, except for the control (0% MD) and the 40% MD. The changes in the particle size of the agglomerated PPSPs were consistent with the SEM image analysis. The magnitudes of the gel strength and viscoelastic moduli (G′ and G″) of the agglomerated PPSPs with 10% MD were higher than those of the control due to the more stable structure formed by better intermolecular interaction in the starch and MD during the agglomeration process. Therefore, our results indicated that the fluidized bed agglomeration process and the MD addition as a binder solution greatly influence the physical, structural, and rheological properties of PPSP.

## 1. Introduction

Starch is widely used in the production of processed food and is one of the most important materials in the food industry. Starch can be used not only as a basic material but also as a food additive, such as a thickener or a stabilizer [1,2]. However, starch cannot be easily hydrated and dissolved in water at room temperature, which limits its use in the food industry [3]. Pregelatinized potato starch (PPS) is a type of modified starch that was created to overcome the limitations of natural starch. As for the starch modification methods, there are mainly physical and chemical modification methods. Recently, consumers have had an antipathy to chemical modification methods as their appetite for healthy and environmental food has increased [4]. Chemical modification is not environmentally friendly, and the possibility of even a small amount of chemical residue in the final food product may limit some applications of this modified starch in the food industry (the recognition of “clean labeling ingredients” is rapidly increasing) [5,6]. Therefore, the physical modification method with which it is possible to overcome these limitations has attracted much attention. PPS is widely used in many foods due to its outstanding viscosity-enhancing properties. Moreover, this material can be easily hydrated even at low temperatures because it has already been processed by heat and gelatinization has occurred during its manufacturing process. In general, pregelatinized starch is produced through spray drying, drum drying, and extrusion processes. Among these procedures, drum drying is the most efficient method and is the most commonly applied technology because it yields homogeneous products [3,7]. PPS can easily penetrate water and heat particles through pre-gelatinization, which greatly facilitates hydration [5]. Therefore, PPS is mainly used in foods that need to be thickened with minimal heat (e.g., instant desserts, instant soups, and dressings), and it has a higher digestion rate than conventional starch. Thus, this ingredient is widely used in baby food and hospital food [2]. 

Pregelatinized potato starch powder (PPSP) is commonly supplied as a fine powder. However, this powder has several disadvantages such as fluttering, excessive cohesiveness, and sticking to container walls due to the electrostatic forces caused by the friction between the fine powder granules [8]. Among them, clumping is the most common phenomenon when used in food; it prevents water penetration and drastically decreases solubility. Therefore, the desired viscosity cannot be easily achieved when using PPSP. The simplest and most reliable method to solve this problem is particle size enlargement through an agglomeration technique that causes structural changes in the starch [9,10]. The fluidized bed agglomeration process (FBAP) not only improves flowability by lowering cohesiveness but also increases the porosity ratio, which in turn increases solubility and dispersibility. Therefore, the FBAP was adopted in this study. The FBAP modifies several characteristics of food powders, including the outer structure, size distribution, flowability, density, wettability, and dispersion behavior [9]. The fluidized bed system favors the formation of homogeneous particles by allowing the particles to move separately. In the FBAP, a liquid binder was sprayed through atomizing nozzles on the fluidized particles, and a hot air stream was formed vertically from below. This hot air stream contributes to the movement of heat and moisture while forming the fluidized bed system in the chamber [11,12,13]. The fluid bed system was created by adjusting the air pressure, air velocity, inlet air temperature, binder feed flow rate (pump rate), and binder concentration. According to the results, the particle size was increased, and the flowability and wetting time were improved [12]. Several studies have demonstrated that the main parameters in the FBAP that can affect the properties of agglomerates include the intrinsic properties of the material itself and the type or concentration of the binder [14,15,16,17,18]. 

To the best of our knowledge, our study is the first to evaluate the effect of particle agglomeration on PPSP. Therefore, in this study, the FBAP was conducted at different concentrations of maltodextrin (MD) (0–40%) as a model binder. The objective of this study was to investigate the changes in the physical (flowability, porosity, dispersibility, solubility, and particle size distribution); structural (scanning electron microscopy); and rheological properties (flow and dynamic rheological properties and gel strength) of PPSP when agglomerated at each binder concentration. Considering these changes, it was possible to determine which MD binder concentration was suitable for the agglomeration of PPSP. Additionally, our findings confirmed the effects of specific MD concentrations on the rheological properties and gel strength of the PPSP solutions.

## 2. Results and Discussion

### 2.1. Particle Size Distribution (PSD)

**Figure 1:**
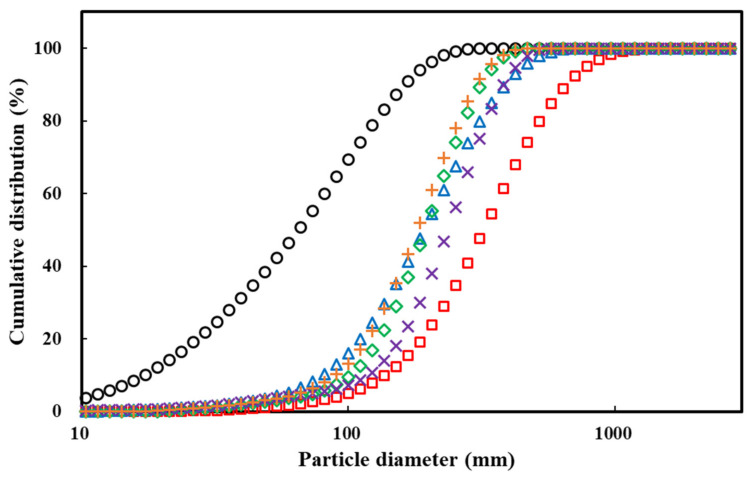
Cumulative distribution of raw and agglomerated PPSPs with different concentrations of MD binder: (○) raw PPSP, (□) PPSP—0%, (△) PPSP—10%, (◇) PPSP—20%, (×) PPSP—30%, and (+) PPSP—40%.

**Figure 2:**
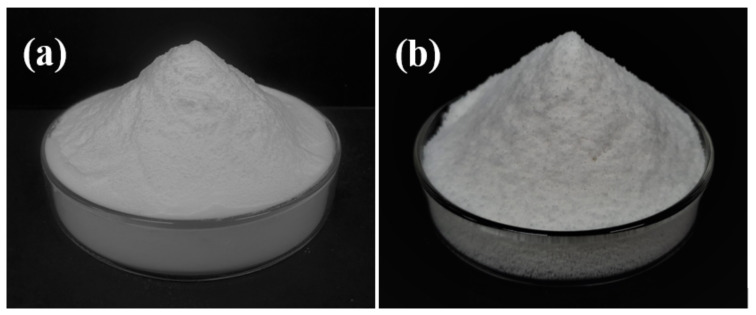
Macrographs of appearance for raw and agglomerated PPSPs: (**a**) raw PPSP and (**b**) PPSP—0%.

**Figure 3:**
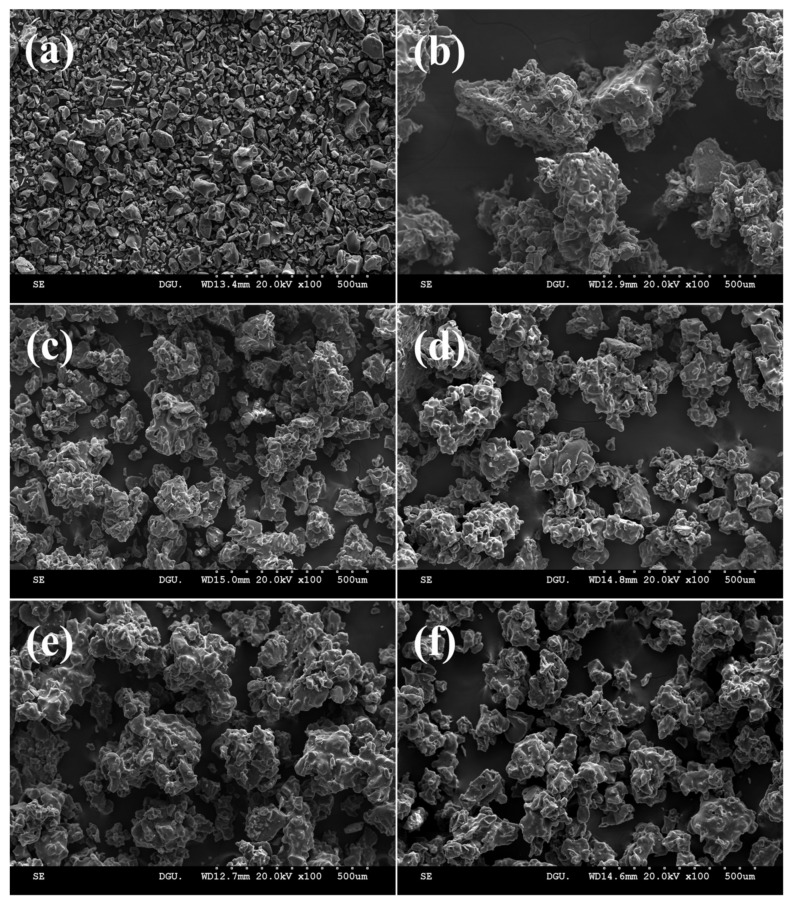
SEM micrographs of raw and agglomerated PPSPs with different concentrations of MD binder: (**a**) raw PPSP, (**b**) PPSP—0%, (**c**) PPSP—10%, (**d**) PPSP—20%, (**e**) PPSP—30%, and (**f**) PPSP—40%. Magnification (100×).

Many physical properties of the powder changed when agglomerated. Among them, the particle size was the first change that could be identified. These size changes affect flowability and density. Therefore, when using powder in the food industry particle size is considered to be a very important characteristic. Figure 1 and Table 1 show the size distribution of the particles and the average diameter of the particles, respectively. As the particle size of the agglomerated powder was significantly larger than that of the raw powder, the difference could be clearly confirmed not only by the naked eye (Figure 2) but also by scanning electron microscope (SEM) (Figure 3). D_10_, D_50_, and D_90_ are the values of the particle diameter at 10%, 50%, and 90% in the cumulative size distribution, respectively. D_10_ and D_90_ are used to calculate the span of the particle size distribution. The particle size of the PPSPs was compared with the D_50_ value, which corresponds to the highest proportion of the particle size distributions with each other. The particle sizes (D_50_) exhibited the following descending order: control (0% MD) > 30% MD > 20% MD > 10% MD > 40% MD > raw powder (Table 1). The PPSP exhibited the largest size when agglomerated with water. This was because the changes in the properties of the material occurred in the pretreatment process, during which excessive heat and friction were applied. The surface of the starch granules that had undergone this process was not intact and was covered with cracks. The sprayed water was in direct contact with amylose in these cracks on the surface, resulting in high viscosity [5]. Therefore, the particle size was increased through a solid bridge made of a water–amylose solution with high viscosity.

When agglomerated using the MD binder, the particle size increased proportionally with the concentration of the MD binder (10–30%), except for 40% MD. This was also due to the strength of the solid bridge, which was proportional to the viscosity [9]. The agglomeration process has a mechanism in which both growing and breakage occur at the same time. When either of them is dominant, the particles are considered to be either growing or fracturing, respectively. Fracturing occurs if the strength of the interparticle bond is less than the sum of the forces considered by the collision (i.e., the frictional force and the weight of the agglomerate). Previous studies have confirmed that agglomerates prepared with different concentrations of various sugar binders have different friability properties [19,20,21]. The span value is used as an indicator of the homogeneity of the powder particles. The lower the span value, the more uniform the particle size. The span value of the agglomerated PPSP at different concentrations was 1.13–1.66, which was more homogeneous than raw PPSP (2.28), indicating that the span range shrank as the MD concentration increased. Therefore, large particles and small particles were present together, and the span range increased [20]. When considering the agglomerated PPSP (40% MD) with the increased proportion of small particles and span in Figure 1 and Table 1, our findings indicated that the agglomerate with 40% MD had less flexibility and increased vulnerability of its rigid bridges when compared to that with 30% MD. The overall experimental results indicated not only that the span range reduced as it was agglomerated with MD but also that the agglomeration process of PPSP was greatly affected by the binder concentration.

### 2.2. Flowability, Cohesiveness, and Porosity (ε)

The values for the Carr index (CI), Hausner ratio (HR), and porosity of the raw and agglomerated PPSPs with MD binder are shown in Table 2. The CI (20–27.4%) and HR (1.26–1.38) of the agglomerated PPSPs were lower than the CI (30.8%) and HR (1.44) of the raw PPSP. This means that the cohesiveness of the powder was lowered and the flowability was improved. In other words, the use characteristics of the powder were improved through the agglomeration process. The HR and CI are indices of the cohesiveness and flowability, respectively. These indices were obtained based on the tapped density and bulk density. The tapped density is determined based on the volume after tapping and is greatly affected by the size, shape, roughness, span, etc., of the powder particles [14,22,23].

The agglomerates prepared with 0% MD, which exhibited the largest particle size, showed HR and CI values that were inadequate even though the particle size was increased. As shown in the SEM image (Figure 3b), these agglomerates exhibited the largest shape angle, the longest shape, and the highest roughness. Additionally, these agglomerates exhibited a wide PSD span compared to the other samples, resulting in poor flowability and cohesiveness (Table 2). When agglomerated with the MD binder, the HR and CI improved as the concentration increased from 0% to 30%. Conversely, the agglomerate with 40% MD showed higher HR and CI values when compared to that with 30% MD. This can be attributed to particle breakage, which can lead to a decrease in size, an increase in small particles, and wider particle size span ranges, as previously discussed.

The ε in agglomerated powders is defined as the fraction of the volume of the voids relative to the total volume (microstructural voids and internal voids). The ε is considered one of the most important properties in a powder sample because it affects the penetration of the solvent (e.g., water), which is related to solubility and dispersibility. This is characterized by an irregular and open structure (making solvent penetration easier), which is accompanied by a high porosity [16,24]. The ε values of the agglomerates (89.6–92.1%) were higher than that (64.4%) of the raw PPSP, as shown in Table 2. Furthermore, our findings confirmed that the agglomerates prepared with different concentrations of MD binders had similar ε ratios, which were slightly higher than that of the control. After agglomeration, the increase in the ε can be attributed to the formation of a larger spherical cluster by the cluster–cluster aggregation of the liquid MD binder and the particles [25]. Therefore, our findings confirmed that the agglomeration process had a great influence on increasing the ε of the PPSP. The MD binder was found to be effective in enhancing the ε and was compatible with MD and starch in the agglomeration process.

### 2.3. Morphology of Powder Particle

The SEM images were used to visualize the shape and structure of the surface of the raw and agglomerated PPSP prepared with MD binders at different concentrations (Figure 3). The raw PPSP particles were small, dense, and heterogeneous (Figure 3a), and the span value of the raw PPSP was higher than those of the other PPSPs (Table 1). Furthermore, the results of our SEM imaging analyses were consistent with the PSD analyses (Table 1). The particle sizes exhibited the following descending order: control > 30% MD > 20% MD > 10% MD > 40% MD > raw. The greater size changes of the control compared to the other agglomerates could be due to the occurrence of high viscosity caused by the contact of amylose with the sprayed water that penetrated into the cracks [5]. These agglomerates exhibited a long shape, which adversely affected flowability. The particle size of the agglomerated PPSPs prepared with MD binder increased as the MD concentration increased from 10% to 30%. In the case of 40% MD, which is the highest MD concentration, the friability of the solid bridge increased and the breaking speed was higher than the growing speed, resulting in a decrease in particle size. The agglomerated PPSPs had a similar spherical shape, indicating that the particles between the same samples became uniform compared to the raw PPSP.

The FBAP makes the particles randomly coalesce through the binder, and therefore, the agglomerates exhibited irregular shapes [26]. As a result, the roughness and porosity increased, as confirmed by our SEM imaging analyses (Figure 3). A previous study demonstrated that an increase in open space promotes water penetration, causing the particles to disperse and dissolve quickly [25]. Based on these findings, we concluded that the FBAP for the preparation of agglomerated PPSP using MD as a binder is suitable for practical use because the food industry requires the quick dissolution and dispersion of powders for the fabrication of instant food.

### 2.4. Powder Dispersibility and Solubility

**Figure 4:**
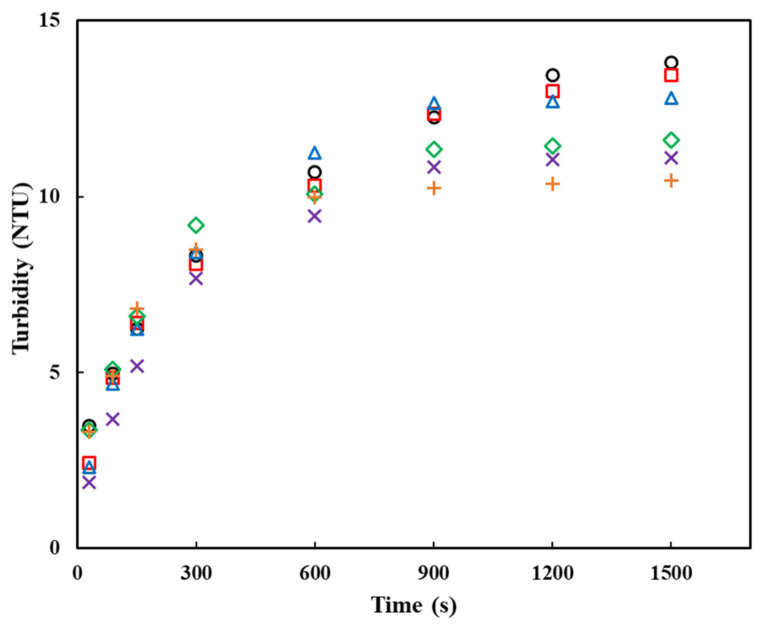
Plots of turbidity versus dissolution time of raw and agglomerated PPSPs with different concentrations of MD binder: (○) raw PPSP, (□) PPSP—0%, (△) PPSP—10%, (◇) PPSP—20%, (×) PPSP—30%, and (+) PPSP—40%.

The powder dispersibility was indirectly observed via turbidimetry as a measure of the suspended or non-dissolved particles in a solution. The turbidity values were acquired by calculating the degree of loss of emitted light due to the blocking of particles. Previous studies reported that the turbidity profile could be used to distinguish between the wetting, swelling, dispersion, and dispersion stages, and that each rate-limiting stage existed according to each material type [11,27]. Therefore, turbidity was applied to measure the dispersibility of the raw and agglomerated PPSPs. The changes in the turbidity of the raw and agglomerated PPSPs were determined over the stirring time (30, 90, 150, 300, 600, 900, 1200, and 1500 s), as shown in Figure 4. The PPSP, which is rarely dissolved in cold water, increased the turbidity and then maintained a constant range as the dispersion progressed. The section in which the turbidity remained constant indicated that the dispersion was completed. The section that was kept constant for the agglomerated PPSPs with MD (10–40%) began at approximately 900 s. In contrast, the turbidity of the raw PPSP and the control continued to increase after 900 s. These turbidity measurements demonstrated that the agglomerated PPSPs with MD had better dispersibility than the raw PPSP and the control. As for the magnitude of the turbidity, the higher the concentration of MD binder, the lower the peak turbidity. This is because the higher the concentration of MD binder, the lower the proportion of starch.

The PPSPs agglomerated through the FBA process had much better solubility than the raw PPSP (Table 2). This can be attributed to the difficulty in achieving dispersion due to the severe clumping in water during stirring for 20 s. The PPSPs agglomerated with MD were slightly better than the control. Other studies have also found that the FBAP improves cluster formation and has high porosity, showing that the structural changes in the powder have a significant impact on solubility and dispersibility [16,25,28]. Accordingly, these results (dispersibility and solubility) can also be related to the ε of PPSP, as shown in Table 2. 

### 2.5. Gel Strength

The gel structure is formed by having a three-dimensional network in which the starch components (amylose and amylopectin) collide with each other. The gel strength is determined by the extent of the intermolecular junction zone [29]. Overall, as the concentration of MD binder increases, the gel strength tends to decrease and increase over the storage time (Table 3). The gel strength of the agglomerated PPSP with 10% MD was stronger than that of the control. In general, it was found that the leaching of amylose decreases when sugar is added [30]. However, amylose leaching can be increased at a sugar concentration of 10–20% for certain types of sugars [31]. Therefore, we can deduce why the gel strength of the agglomerated PPSP with 10% MD increased. It is worth noting that the gel strength of the agglomerates with 10% MD was stronger even though the starch content was lower than that of the control. From these results, it was found that MD in a specific concentration range interacts with the starch, and the structure of the starch can be stable in the agglomeration process. The gel strength of the raw PPSP was weaker than those of the agglomerates, showing that the gel structure with desired strength was not formed due to poor reconstitution properties. The gel strength values of the fine and agglomerated PPSPs increased with an increase in storage time, resulting in increased retrogradation over time. However, there was no significant difference in the rate of retrogradation during storage time between the samples with different MD concentrations. Understanding the gel strength of starch gels is important in predicting the physical properties of starch gels when starches are applied to food.

### 2.6. Rheological Properties

**Figure 5:**
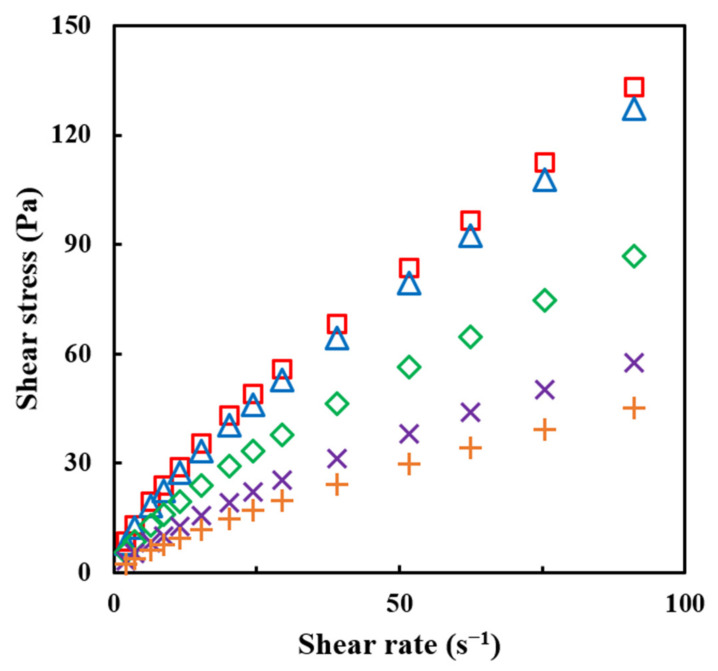
Plots of shear stress versus shear rate for raw and agglomerated PPSPs with different concentrations of MD binder: (○) raw PPSP, (□) PPSP—0%, (△) PPSP—10%, (◇) PPSP—20%, (×) PPSP—30%, and (+) PPSP—40%.

The data on the shear stress and shear rate follow the power law model with a high coefficient of determination (0.99–1.00) (Figure 5 and Table 4). Therefore, the flow behavior of the agglomerated PPSP samples was presented as the values of K, n, and η_a,50_ acquired from the power-law equation (Equation (7)). The viscosity is the resistance applied to a liquid, which increases as the length of the chain increases and is proportional to the number of chains [29]. The viscosity indices (K and η_a,50_) tend to decrease as the concentration of MD increases. This is because the ratio of the starch components of the sample decreases when the concentration of MD increases. In other words, the number of long chains (starch component) in the solution decreases because the long-chain molecules were replaced with the short-chain (MD) through the FBAP. Therefore, the resistance in the liquid decreases, and thus, the viscosity decreases. The n value is used as an index for the flow behavior of the fluid. All the samples have values of n < 1. This means that the samples exhibited the characteristics of a pseudoplastic fluid. In other words, the fluid with high pseudoplasticity (n close to 0) flows better than the fluid with low pseudoplasticity (close to 1) because it has a lower viscosity at the same shear rate [32]. In this study, the n value for the agglomerated PPSP tends to increase as the concentration of the MD binder increases from 10% to 40%. This means that the viscosity at 50 s^−1^ decreases as the MD concentration increases. 

**Figure 6:**
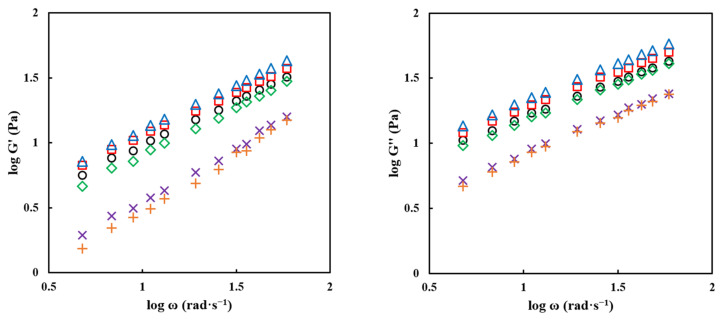
Plots of log G′ and log G″ versus log ω for raw and agglomerated PPSPs with different concentrations of MD binder: (○) raw PPSP, (□) PPSP—0%, (△) PPSP—10%, (◇) PPSP—20%, (×) PPSP—30%, and (+) PPSP—40%.

The viscoelastic moduli (G′ and G″) generally tended to decrease as the MD concentration increased, as shown in Figure 6 and Table 5. However, the PPSP agglomerated at 10% MD had a higher magnitude of viscoelastic moduli than that of the control. This can be attributed to better intermolecular interaction between starch and MD during the agglomeration process. These results were also confirmed by the gel strength measurements (Table 3). These observations demonstrated that MD not only affected the gel state but also the liquid state. Therefore, our findings demonstrated that the PPSP with 10% MD is the optimal combination when agglomerating, showing a structure with great rigidity and elasticity. Additionally, the results of the magnitudes of G′ and G″ of a solution made of the agglomerated PPSPs (30% and 40%) were significantly reduced when used at a concentration higher than 20% MD. These results demonstrated that the matrix structure formed by a collision between chains was rapidly weakened due to the low starch content. Moreover, our findings demonstrated that MD did not have a significant impact on these matrices. Our results also demonstrated that tan δ tended to increase significantly as the concentration of MD binders increased. These results suggest that the viscous behavior gradually becomes dominant as the concentration of the MD binder increases [14], indicating that the liquid property of the solution increased significantly. From the overall results, it can be concluded that the rheological properties of the agglomerated PPSPs were greatly affected by the concentration of MD. The viscosity and viscoelastic moduli of the raw PPSP were much lower than those of the control. This can be attributed to the small lumps, which were due to the poor reconstitution property of the raw powder. 

## 3. Conclusions

This study discusses the impact of agglomerating pregelatinized potato starch powder (PPSP) with different concentrations of maltodextrin (MD) through a fluidized bed agglomeration process (FBAP). The raw and agglomerated PPSPs were investigated and compared with each other, and our findings confirmed that various properties (physical, morphological, reconstitution, and rheological properties) changed. PPSP is supplied in the form of a fine powder, which results in flowability and dispersibility issues. Therefore, the FBAP was adopted to improve these properties. The FBAP increased the size of the PPSP while decreasing the size distribution span, resulting in better flowability. However, in the case of PPSP with 40% MD, the rate of breaking was more dominant due to its high friability, resulting in a decrease in size and an increase in span, in addition to a decrease in flowability. Furthermore, our findings demonstrated that the FBAP resulted in irregularly shaped PPSP particles while increasing their size and porosity, resulting in increased solubility and dispersibility. Additionally, given the lack of published experimental data on the rheological properties and gel strength of agglomerated starch, our study characterized the rheology and gel strength of agglomerated PPSPs using MD as a binder. In the case of PPSP agglomerated with 10% MD, the viscosity was lowered because the number of chains in the solution was less than that of the PPSP agglomerated with 0% MD. However, the magnitudes of the viscoelastic moduli (G′ and G″) were increased due to the strengthening of the structure by MD. This tendency was also observed in the gel strength measurements. These findings indicated that the fluidized bed agglomeration process and the MD addition as a binder solution greatly influenced the physical, structural, and rheological properties of PPSP.

This is the first study that evaluates the impact of FBAP with different concentrations of MD on PPSP. The FBAP for the preparation of agglomerated PPSP samples was successfully performed, and their properties were improved. Therefore, understanding the gel strength and rheological properties of PPSP is important for predicting the physical properties of the gel or the solution state when starches are applied in the food industry. The knowledge of these specific properties of agglomerated PPSPs prepared with MD binder would be useful in developing high value-added foods, such as silver food, baby food, nutritional supplies, cosmetics, etc. Further studies on various types of sugar binders with different concentrations are needed to expand the results of this study.

## 4. Materials and Methods

### 4.1. Materials

Commercial PPS (Avebe, Veendam, The Netherlands) was used for producing agglomerates in a fluidized bed system. Its approximate compositions were: 7.6% moisture content, 0.05% protein, 0.05% fat, 0.3% ash, and 92% carbohydrate. MD with DE14 (Daesang Corp, Gun-San, Republic of Korea) was added as a standard binder to the FBAP.

### 4.2. FBAP

Agglomeration was conducted using a top-spray fluidized bed agglomerator (Fluid Bed Lab System, Dae Ho Technology Co., Ltd., Hwaseong, Republic of Korea) [14]. The MD was thoroughly dissolved in distilled water at room temperature to obtain various MD concentrations ranging from 10% to 40% (*w*/*w*). For comparison, a binder solution with no added MD (i.e., only deionized water) was also used to prepare an agglomerated PPS sample as a control. The original fine PPS powder was first deposited in the product container, after which an upward-flowing hot air stream was used to create the fluidized bed system inside. The binder (1000 mL for PPS, proceeding for 40 min) was then supplied by a peristaltic pump at a 25 mL/min flow rate and sprayed with fine droplets through a spray nozzle onto the flowing powder with a spray pressure of 1.5 bar. The inlet air temperature was set at 78 ± 1.0 °C. Throughout the spraying operation, the product temperature was kept constant at 53 ± 1.0 °C. The damper and blower were preset at 30% and 80%, respectively. After spraying with binder solution, the product was cooled down and dried for 10 min at room temperature using fluidizing air.

### 4.3. Particle Size Distribution Measurements

Particle size distribution (PSD) was determined using a Malvern Mastersizer (Mastersizer 3000E, Malvern Instruments Ltd., Worcestershire, UK). This device measures the particle size of the powder through light refraction [33]. The D_10_, D_50_, and D_90_ mean of the cumulative particle diameters represent 10%, 50%, and 90% of the entire range of size. The span index was calculated with Equation (1):

(1) $$\mathrm{Span}=\frac{{(D}_{90}-{\;D}_{10})}{D_{50}}$$

### 4.4. Flowability and Cohesiveness Measurements

The Hausner ratio (HR) [34] and Carr index (CI) [35] values were calculated to assess the extent of the cohesiveness and flowability of a powder, respectively. After pouring powder (20 g) into a 100 mL glass mass cylinder, it was tapped 1250 times using a tap density tester (BT-301, K-ONE Ltd., Seoul, Republic of Korea). The bulk (ρ_bulk_) and tapped density (ρ_tapped_) were obtained by dividing the mass of the filled powder by the volume occupied by the powders before and after being tapped, respectively. Once the ρ_bulk_ and ρ_tapped_ were calculated, the HR and CI could be calculated, respectively, with Equations (2) and (3) below:

(2) $$\mathrm{HR}=\frac{\rho_{\mathrm{tapped}}}{\rho_{\mathrm{bulk}}}$$

(3) $$\mathrm{CI}=\frac{\left(\rho_{\mathrm{tapped}}-\rho_{\mathrm{bulk}}\right)}{\rho_{\mathrm{tapped}}}\times 100$$

The cohesiveness of the powder is considered to be low when HR < 1.2, intermediate when 1.2 < HR < 1.4, and high when HR > 1.4. Furthermore, the flowability of a powder is considered very good when CI < 15, good when 15 < CI < 20, fair when 20 < CI < 35, bad when 35 < CI < 45, and very bad when CI > 45.

### 4.5. Particle Density (ρ_particle_) and Porosity (ε) Measurements

To acquire the ρ_particle_ values, a 10 mL glass mass cylinder was first filled with the powder (0.5 g), after which 5 mL of petroleum ether was added to form a suspension that occupied the vacant spaces within the particles. Pipetting was performed to promote the penetration of the solvent into the porous space of the particles. Then, 1 mL of additional petroleum ether was added to rinse off the powder that had been attached to the cylinder wall due to pipetting. After making the suspension, ρ_particle_ was calculated with Equation (4):

(4) $$\rho_{\mathrm{particle}}=\frac{W_{P}}{V_{t}-6}$$

where W_p_ is the mass of the powder (g), and V_t_ is the total volume of the suspension (mL). The porosity (ε) of the powder was then calculated with Equation (5):

(5) $$\varepsilon \;=\frac{\rho_{\mathrm{particle}}-\rho_{\mathrm{tapped}}}{\rho_{\mathrm{particle}}}\;\times \;100\;(\%)$$

### 4.6. Scanning Electron Microscopy (SEM)

The powders were vacuum-coated with platinum-palladium after being mounted to an aluminum stub with double-sided adhesive carbon tape [11]. Particle morphology was assessed using images taken at 100× magnification using SEM (Hitachi S-3000 N SEM, Hitachi Ltd., Tokyo, Japan), running at 20 kV.

### 4.7. Powder Dispersibility

The turbidity of the powder dispersed in the water was used to assess dispersibility. First, 5 mg of agglomerates was poured into 100 mL of distilled water, which was promptly agitated to disperse the fine PPS powder and the agglomerates. Then, the turbidity of the suspension was determined with a turbidimeter (AQ4500, Thermo Fisher Scientific Inc., Waltham, MA, USA) after agitating for 30 s, 90 s, 150 s, 300 s, 600 s, 900 s, 1200 s, and 1500 s, respectively.

### 4.8. Solubility

Solubility was estimated as described by Bello-Perez et al. [36]. A PPS solution (3%, *w*/*v*) was made by constant stirring for 20 s to ensure dispersal. To gelatinize the starch, the solution was transferred to a water bath maintained at 80 °C and shaken intermittently every 10 min, after which the solution was left undisturbed for 60 min. The starch solution was then cooled to room temperature in ice water. Next, the suspension was centrifuged at 4000 rpm for 20 min. The separated supernatant was transferred to a weighed beaker and then dried overnight in a dryer at 105 °C. Afterwards, the soluble starch residue was weighted, and the solubility was calculated with Equation (6):

(6) $$\mathrm{Solubility}\;\left(\%\right)=\frac{W_{s}}{W_{d}}\;\times \;100(\%)$$

where W_s_ is the weight of the PPS in the supernatant (g), and W_d_ is the weight of the PPS in the suspension (g).

### 4.9. Gel Strength

All the samples were prepared immediately before each measurement. Each solution was prepared by dissolving fine PPS and agglomerated powders in distilled water at a concentration of 15% (*w*/*w*) with constant stirring. Each solution was stirred for 1 h in a water bath at 95 °C for 60 min to be fully gelatinized. After taping the circumference of the cylinder with a diameter of 35 mm and height of 40 mm, the solution was slowly poured into the cylinder to prevent air bubbles. After cooling it at room temperature for 1 h, the cylinder was sealed to prevent moisture evaporation and left at 4 °C for 3, 5, and 7 days. After removing the tape, the uneven surface was cut to make it smooth, after which the measurements were conducted.

Gel strength measurements were conducted using a texture analyzer (Brookfield Texture Analyzer, TA-CT3, Brookfield, Middleboro, MA, USA). Moreover, using a 10 mm diameter probe, the penetration speed and depth were set to 1 mm/s and 10 mm, respectively. Hardness (g_f_), which is defined as the force required to compress the gel, was also determined.

### 4.10. Rheological Properties

The samples were prepared in the same manner as in the gel strength measurement except for the cooling step. Afterwards, each hot solution was immediately transferred onto the plate of a Haake RheoStress 1 rheometer (Haake GmbH, Karlsruhe, Germany) for measurement. Its flow and dynamic rheological properties were determined as described by Lee and Yoo [11]. Both experiments used a plate–plate geometry with a 35 mm diameter, a plate temperature of 25 °C, and a gap of 500 μm between the plate and the probe. The shear stress of the solution was measured at a shear rate range of 0.1–100 s^−1^ to investigate the rheological characteristics of the agglomerates. The experimental results were then substituted into the power-law equation:

(7) $$\sigma =K\;{\dot{\gamma }}^{n}$$

where σ is the shear stress (Pa), $\dot{\gamma }$ is the shear rate (s^−1^), and n is the flow behavior index. The consistency index (K) and the apparent viscosity at 50 s^−1^ (η_a,50_) were calculated with Equation (7). The dynamic rheological behaviors of the agglomerates were determined through a frequency sweep test at an angular frequency range of 0.63–62.8 rad·s^−1^ (0.1–10 Hz) with a 2% strain, which is the linear viscoelasticity limit of the sample. Based on these measurements, the storage (or elastic) modulus (G′), the loss (or viscous) modulus (G″), and the loss tangent (tan δ) of the agglomerate at 6.28 rad·s^−1^ were calculated using the Haake Rheowin software (ver. 4.50.0003), after which we compared the differences in viscoelasticity between the agglomerates.

### 4.11. Statistical Analysis

All the experiments were conducted in triplicate. The physical and rheological parameters are presented as the mean ± standard deviation. Duncan’s multiple range tests were used to compare the samples using SAS version 9.4 (SAS Institute, Cary, NC, USA). The level of statistical significance was set at *p* < 0.05.

## Figures and Tables

**Table 1:** Particle size distribution (PSD) of raw and agglomerated PPSPs with different concentrations of MD binder.

Sample	MD Concentration (%)	D_10_ (μm)	D_50_ (μm)	D_90_ (μm)	Span
Raw PPSP		17.2 ± 0.01 ^f^	64.8 ± 0.07 ^e^	165 ± 1.39 ^f^	2.28 ± 0.02 ^a^
Agglomerated PPSP	0 (control)	133 ± 3.00 ^a^	311 ± 7.93 ^a^	637 ± 12.5 ^a^	1.62 ± 0.01 ^c^
	10	80.0 ± 0.91 ^e^	191 ± 1.08 ^c^	395 ± 3.35 ^b^	1.66 ± 0.02 ^b^
	20	104 ± 1.57 ^c^	195 ± 3.52 ^c^	331 ± 4.07 ^d^	1.17 ± 0.02 ^d^
	30	116 ± 0.59 ^b^	234 ± 1.21 ^b^	380 ± 1.14 ^c^	1.13 ± 0.00 ^e^
	40	88.4 ± 1.30 ^d^	179 ± 1.39 ^d^	300 ± 1.89 ^e^	1.18 ± 0.01 ^d^

Each value is the mean of triplicate measurements ± SD. Means with different lowercase letters (a–f) within each column are significantly different (*p* < 0.05).

**Table 2:** Flow characteristics, porosity (ε), and solubility of raw and agglomerated PPSPs with different concentrations of MD binder.

Sample	MD Concentration (%)	ρ_bulk_ (g/cm^3^)	ρ_tapped_ (g/cm^3^)	ε (%)	CI (%)	HR	Solubility (%)
Raw PPSP		0.62 ± 0.01 ^a^	0.89 ± 0.02 ^a^	64.4 ± 0.79 ^c^	30.8 ± 0.48 ^a^	1.44 ± 0.00 ^a^	31.9 ± 1.02 ^d^
Agglomerated PPSP	0 (control)	0.22 ± 0.01 ^e^	0.31 ± 0.00 ^e^	89.6 ± 0.95 ^b^	27.4 ± 0.11 ^b^	1.38 ± 0.00 ^b^	93.1 ± 0.69 ^c^
	10	0.33 ± 0.01 ^bc^	0.46 ± 0.00 ^b^	91.1 ± 2.31 ^ab^	27.2 ± 0.31 ^b^	1.37 ± 0.00 ^c^	96.0 ± 0.88 ^b^
	20	0.29 ± 0.01 ^d^	0.39 ± 0.01 ^d^	91.3 ± 0.79 ^ab^	24.7 ± 0.40 ^c^	1.33 ± 0.01 ^d^	97.5 ± 0.40 ^a^
	30	0.35 ± 0.01 ^b^	0.44 ± 0.01 ^c^	91.3 ± 1.75 ^ab^	20.8 ± 0.96 ^d^	1.26 ± 0.01 ^e^	95.8 ± 0.68 ^b^
	40	0.33 ± 0.00 ^c^	0.43 ± 0.01 ^c^	92.1 ± 1.51 ^a^	24.7 ± 0.59 ^c^	1.33 ± 0.01 ^d^	96.6 ± 0.51 ^ab^

Each value is the mean of triplicate measurements ± SD. Means with different lowercase letters (a–e) within each column are significantly different (*p* < 0.05).

**Table 3:** Gel strength of raw and agglomerated PPSPs with different concentrations of MD binder over storage time.

Sample	MD Concentration (%)	Gel Strength (g_f_)		
		3 Days	5 Days	7 Days
Raw PPSP		9.67 ± 0.58 ^d^	40.7 ± 2.31 ^d^	108 ± 4.16 ^d^
Agglomerated PPSP	0 (control)	21.7 ± 1.53 ^b^	66.3 ± 1.15 ^b^	161 ± 1.53 ^b^
	10	32.3 ± 1.15 ^a^	76.7 ± 0.58 ^a^	177 ± 3.79 ^a^
	20	21.7 ± 2.08 ^b^	56.3 ± 1.53 ^c^	143 ± 2.31 ^c^
	30	12.0 ± 1.00 ^c^	43.0 ± 1.00 ^d^	111 ± 1.73 ^d^
	40	9.00 ± 1.00 ^e^	26.3 ± 0.58 ^e^	100 ± 1.00 ^e^

Each value is the mean of triplicate measurements ± SD. Means with different lowercase letters (a–e) within each column are significantly different (*p* < 0.05).

**Table 4:** Values of power law model parameters (η_a,50_, K, and n) of raw and agglomerated PPSPs with different concentrations of MD binder.

Sample	MD Concentration (%)	η_a,50_ (Pa·s)	K (Pa·s^n^)	n
Raw PPSP		1.47 ± 0.02 ^c^	4.78 ± 0.17 ^ab^	0.70 ± 0.01 ^d^
Agglomerated PPSP	0 (control)	1.67 ± 0.02 ^a^	4.89 ± 0.01 ^a^	0.73 ± 0.00 ^c^
	10	1.58 ± 0.06 ^b^	4.70 ± 0.02 ^b^	0.72 ± 0.01 ^c^
	20	1.10 ± 0.02 ^d^	3.18 ± 0.10 ^c^	0.73 ± 0.00 ^c^
	30	0.76 ± 0.01 ^e^	1.45 ± 0.07 ^d^	0.77 ± 0.00 ^b^
	40	0.59 ± 0.00 ^f^	1.33 ± 0.07 ^d^	0.79 ± 0.01 ^a^

Each value is the mean of triplicate measurements ± SD. Means with different lowercase letters (a–f) within each column are significantly different (*p* < 0.05).

**Table 5:** Values of dynamic rheological parameters (G′, G″, and tan δ) of raw and agglomerated PPSPs with different concentrations of MD binder.

Sample	MD Concentration (%)	G′ (Pa)	G″ (Pa)	tan δ
Raw PPSP		6.71 ± 0.05 ^c^	12.1 ± 0.01 ^c^	1.80 ± 0.02 ^d^
Agglomerated PPSP	0 (control)	8.13 ± 0.03 ^b^	14.3 ± 0.08 ^b^	1.76 ± 0.00 ^e^
	10	8.68 ± 0.04 ^a^	15.8 ± 0.03 ^a^	1.82 ± 0.01 ^d^
	20	5.48 ± 0.03 ^d^	11.1 ± 0.06 ^d^	2.02 ± 0.00 ^c^
	30	2.27 ± 0.04 ^e^	6.15 ± 0.04 ^e^	2.71 ± 0.04 ^b^
	40	1.86 ± 0.03 ^f^	5.70 ± 0.08 ^f^	3.07 ± 0.03 ^a^

Each value is the mean of triplicate measurements ± SD. Means with different lowercase letters (a–f) within each column are significantly different (*p* < 0.05).

## Data Availability

All the results shown in the manuscript could be requested from the corresponding author, who would provide them.
